# Lyophilized powder of mesenchymal stem cell supernatant attenuates acute lung injury through the IL-6–p-STAT3–p63–JAG2 pathway

**DOI:** 10.1186/s13287-021-02276-y

**Published:** 2021-03-29

**Authors:** Wenjun Peng, Meijia Chang, Yuanyuan Wu, Wensi Zhu, Lin Tong, Ge Zhang, Qin Wang, Jie Liu, Xiaoping Zhu, Tingting Cheng, Yijia Li, Xi Chen, Dong Weng, Sanhong Liu, Hongwei Zhang, Yao Su, Jian Zhou, Huayin Li, Yuanlin Song

**Affiliations:** 1 Department of Pulmonary and Critical Care Medicine, Shanghai Respiratory Research Institute, Zhongshan Hospital, Fudan University, Shanghai, 200032 China; 2 Department of Pulmonary and Critical Care Medicine, Shanghai East Hospital, Tongji University School of Medicine, Shanghai, 200120 China; 3 Public Translational Platform for Cell Therapy, Yangtze Delta Region Institute of Tsinghua University, Hangzhou, 311200 Zhejiang China; 4 Yunnan Province Stem cell Bank, Kunming, 650101 Yunnan China; 5 Department of Pulmonary and Critical Care Medicine, Shanghai Pulmonary Hospital, Tongji University School of Medicine, Shanghai, 200433 China; 6 Institute of Interdisciplinary Integrative Medicine Research, Shanghai University of Traditional Chinese Medicine, Shanghai, 201203 China; 7 Center of Emergency & Intensive Care Unit, Jinshan Hospital, Fudan University, Shanghai, 200540 China

**Keywords:** Acute lung injury, Bleomycin, Mesenchymal stem cells, Secretome, Lyophilized powder, p63

## Abstract

**Background:**

Acute lung injury (ALI) and acute respiratory distress syndrome (ARDS) are syndromes of acute respiratory failure with extremely high mortality and few effective treatments. Mesenchymal stem cells (MSCs) may reportedly contribute to tissue repair in ALI and ARDS. However, applications of MSCs have been restricted due to safety considerations and limitations in terms of large-scale production and industrial delivery. Alternatively, the MSC secretome has been considered promising for use in therapeutic approaches and has been advanced in pre-clinical and clinical trials. Furthermore, the MSC secretome can be freeze-dried into a stable and ready-to-use supernatant lyophilized powder (SLP) form. Currently, there are no studies on the role of MSC SLP in ALI.

**Methods:**

Intratracheal bleomycin was used to induce ALI in mice, and intratracheal MSC SLP was administered as a treatment. Histopathological assessment was performed by hematoxylin and eosin, immunohistochemistry, and immunofluorescence staining. Apoptosis, inflammatory infiltration, immunological cell counts, cytokine levels, and mRNA- and protein-expression levels of relevant targets were measured by performing terminal deoxynucleotidyl transferase dUTP nick-end labeling assays, determining total cell and protein levels in bronchoalveolar lavage fluids, flow cytometry, multiple cytokine-detection techniques, and reverse transcriptase-quantitative polymerase chain reaction and western blot analysis, respectively.

**Results:**

We found that intratracheal MSC SLP considerably promoted cell survival, inhibited epithelial cell apoptosis, attenuated inflammatory cell recruitment, and reversed immunological imbalances induced by bleomycin. MSC SLP inhibited the interleukin 6–phosphorylated signal transducer and activator of transcription signaling pathway to activate tumor protein 63–jagged 2 signaling in basal cells, suppress T helper 17 cell differentiation, promote p63^+^ cell proliferation and lung damage repair, and attenuate inflammatory responses.

**Conclusions:**

MSC SLP ameliorated ALI by activating p63 and promoting p63^+^ cell proliferation and the repair of damaged epithelial cells. The findings of this study also shed insight into ALI pathogenesis and imply that MSC SLP shows considerable therapeutic promise for treating ALI and ARDS.

**Supplementary Information:**

The online version contains supplementary material available at 10.1186/s13287-021-02276-y.

## Background

Acute lung injury (ALI) and its most severest manifestation, acute respiratory distress syndrome (ARDS), are characterized by clinical hypoxemia combined with bilateral pulmonary infiltration and edema [1]. Over the past two decades, the mortality rate of ARDS has remained high at around 40% with no trend of decreasing, resulting in a major health-care burden in both developing and developed countries [2, 3]. Current treatment is primarily supportive and focuses on treating the underlying cause and triggers of ALI and ARDS [4]. Although lung-protective ventilation can improve airway pressure to a certain extent and result in improvement of lung function and decreased mortality, no specific treatment has been demonstrated to be satisfactory. In a clinical trial, mechanical ventilation with different levels of positive end-expiratory pressure only reduced the 28-day mortality of ALI patients to approximately 30%, which still remained quite high [5]. Therefore, efficient pharmaceuticals and specific treatments are urgently needed.

Mesenchymal stem cells (MSCs) are multipotent cells that have attracted much attention due to their potential in regenerating defective tissues and their immunomodulatory capabilities in inflammatory disorders [6]. MSCs were initially found in the bone marrow and then isolated from most postnatal organs [7], such as adipose tissue [8], the lungs [9], and tangential blood [10]. Fetal tissues, including the placenta and umbilical cord, are also available sources of MSCs [11]. Over the past decades, MSCs and the MSC secretome have shown great promise for treating a great assortment of diseases, especially severe disorders without effective pharmacotherapies [12, 13]. Previously, MSC therapy showed good efficacy against ALI, based on the potential of MSC colonization and differentiation, and the ability of MSCs to secrete soluble bioactive molecules [14, 15]. However, MSCs are inconvenient to produce and store, and the risks of iatrogenic tumor formation and pulmonary embolism remain as concerns for MSC therapy [16]. Because MSC engraftment is transient, much of the current research related to MSC therapy has focused on the paracrine-signaling capacity and the MSC secretome. In this study, the secretome of placenta-derived MSCs was collected and purified from culture supernatants and then freeze-dried into an MSC supernatant lyophilized powder (MSC SLP) form. The specific procedure for preparing MSC SLP is presented in Supplementary Fig. 1.

CD4^+^CD25^+^Foxp3^+^ regulatory T (Treg) and T helper 17 (Th17) cells play a prominent role in the immune system and are implicated in host defense and a large number of autoimmune and inflammatory diseases [17], including ALI and ARDS [18, 19]. Clinically, Treg cells are often found to be significantly increased in patients with ARDS and are associated with mortality [19, 20]. Th17 cells represent a pro-inflammatory subset compared to Treg cells [21]. In ARDS, Th17 cells recruit pro-inflammatory cytokines and aggravate the inflammatory response [19, 20].

Interleukin-6 (IL-6), which is primarily secreted by macrophages and type-2 pneumocytes in the lungs, has been commonly identified as a vital pro-inflammatory cytokine involved in various inflammatory disorders of the lungs [22–25]. IL-6 has also been implicated in the pathogenesis of bleomycin (BLM)-induced inflammatory injury and subsequent fibrosis [26, 27]. At the inflammatory stage, BLM-induced IL-6 is produced predominantly by type-2 pneumocytes and functions as a pro-inflammatory and anti-fibrosis factor [27], and IL-6 deficiency attenuates inflammatory cell recruitment [26]. At the fibrotic stage, BLM-induced IL-6 is produced mainly by macrophages and fibroblasts and has pro-fibrotic activity [27].

Basal cells are multipotent tissue-specific epithelial progenitors that express tumor protein 63 (p63, also known as Trp63 and TP63), cytokeratin 5 (Krt5), and cytokeratin 14 (Krt14). They function as stem cells that proliferate rapidly following epithelial injury and help regenerate damaged epithelium in both mouse trachea and human airways [28, 29]. Normally, basal cells are relatively quiescent, as they can rapidly self-renew and differentiate in response to stimulation [28]. As a member of the p53 family, p63 is highly expressed in basal cells of various epithelial tissues and confers stem cell properties [30, 31]. Similarly, p63 has been shown to be a vital mediator of normal development, maintenance, and homeostasis of the epithelium [30, 32]. In addition, p63 has been widely documented in various epithelial tumors, including squamous cell carcinomas of the lungs, prostate, and bladder [33, 34].

BLM is a chemotherapeutic drug used against various human malignancies and also widely applied in murine models to induce ALI at the inflammatory phase (within 1 week) or pulmonary fibrosis at the fibrotic phase (during the second week) [35, 36]. In this study, we established a mouse model of BLM-induced ALI and compared its distinct characteristics before and after MSC SLP treatment to evaluate the mechanism by which MSC SLP attenuates ALI.

## Materials and methods

### MSC SLP preparation

Placenta-derived MSCs were isolated from human placental chorion samples in the Yunnan Province Stem Cell Bank (Yunnan, China). The protocol for collection of umbilical cord and placenta for scientific research was approved by the Health Commission of Yunnan Province. After obtaining informed consent from potential donors, the donated umbilical cord and placenta were collected and then transported to the Yunnan Province Stem Cell Bank. Chorion tissue blocks were cultured in α-MEM with 7% human platelet lysate (UltraGRO-Advanced, AventaCell Biomedical, Atlanta, GA, USA). Approximately 7–10 days later, the primary cells could be passaged to passage 1 (P1). When the P1 cell density reached 80%, the culture could be passaged to P2. In the same manner, the cells were passed from P2 to P3. When the P3 cell density reached approximately 70%, placenta-derived MSCs were washed three times with phosphate-buffered saline (PBS) and cultured in human platelet lysate-free α-MEM medium for 24 h. Then, supernatants from placenta-derived MSCs were collected and processed into lyophilized powder. The procedure used is shown in detail in Supplementary Fig. 1. MSC SLP was sealed and stored at − 20 °C for later use.

### Animals

Eight-week-old male C57BL/6 mice were routinely bred in the animal facility of the Zhongshan Hospital at Fudan University (Shanghai, China). The mice were housed at 20–25 °C with a relative humidity of 50–70% and were provided free access to water and food. The experimental procedures were approved by the Animal Care and Use Committee of Zhongshan Hospital at Fudan University. Intratracheal BLM (2.5 mg/kg) was instilled in mice to induce ALI, and mice in the control group received an equal volume of PBS. MSC SLP (50 mg/kg) was dissolved in PBS and administered intratracheally 1 h after BLM instillation. The mice were sacrificed to collect peripheral blood, bronchoalveolar lavage fluids (BALFs), and lung tissues on day 7. To evaluate the role of IL-6 in ALI, recombinant human (rh) IL-6 (PeproTech, #200-06, NJ, USA) was administrated intratracheally at a dose of 1000 ng per mouse. MSC SLP (50 mg/kg), dissolved in PBS, was also administered intratracheally 1 h after rh IL-6 instillation, and the mice were sacrificed on day 2.

### Hematoxylin and eosin (H&E) staining

The right upper lobes of the lung samples were harvested, fixed in 4% paraformaldehyde overnight, and embedded in paraffin. Lung sections were stained with H&E, and pathological-damage scores were calculated to assess lung injury as follows: no injury = 0, injury in less than 25% of the field = 1, injury in 25–50% of the field = 2, injury in 50–75% of the field = 3, and injury in more than 75% of the field = 4. Ten fields were randomly selected and assessed by investigators blinded to the grouping.

### Total protein concentrations and inflammatory cell counts in BALFs

BALFs were collected by cannulating the trachea and centrifuged at 1000 rpm for 5 min at 4 °C. Each supernatant was stored at − 80 °C, and each cell pellet was resuspended and stained with Wright–Giemsa staining solution (Thermo Fisher Scientific, Waltham, MA, USA). Cell-free supernatants were used to measure total protein levels with the Bicinchoninic Acid (BCA) Protein Assay Kit (7780, Cell Signaling Technology [CST], Boston, MA, USA).

### Myeloperoxidase (MPO) activity determination

The left lungs of mice were weighted and ground into tissue homogenates. MPO activity in lung homogenates was determined using the MPO Assay Kit (ab105136, Abcam, Cambridge, MA, USA), following the manufacturer’s instructions.

### Flow cytometry

The harvested lungs were minced mechanically, digested with an enzyme mix (buffer S, enzyme D, and enzyme A from Miltenyi Biotec, Bergisch Gladbach, Germany), washed, and re-suspended in PBS. Heparin, antibodies, and red blood cell lysis buffer were sequentially added to whole blood samples. Cells were stained with a fluorescein isothiocyanate (FITC)-conjugated antibody against cluster of differentiation (CD) protein 4, an allophycocyanin (APC)-conjugated antibody against CD25, and a phycoerythrin-conjugated antibody against forkhead box P3 (Foxp3) to detect Treg cells. In addition, staining with CD3-FITC and CD8-APC antibodies was performed to detect CD4^+^ T cells (CD3^+^CD8^−^ T cells), and intracellular staining against IL-17A was performed to detect Th17 cells. The antibodies were purchased from eBioscience (San Diego, CA, USA), and staining was performed according to the manufacturer’s instructions.

### Apoptosis detection

To identify apoptotic cells, terminal deoxynucleotidyl transferase dUTP nick-end labeling (TUNEL) assays were performed using an In Situ Cell Death Detection Kit (Roche, Indianapolis, IN, USA). All procedures were performed in accordance with the manufacturer’s instructions.

### Immunofluorescence staining

Lung sections were incubated into 5% goat serum to block nonspecific binding, followed by incubation with primary antibodies overnight at 4 °C. Primary antibodies against p63 (ab53039, Abcam), alpha smooth muscle actin (α-SMA; Servicebio, Wuhan, China), and Ki-67 (ab15580, Abcam) were used. After washing the sections three times in PBS (10 min each wash), they were incubated with an appropriate secondary antibody for 2 h in the dark at 21 °C. The secondary antibodies used included an Alexa Fluor 488-conjugated goat anti-rabbit IgG (4412, CST), a cyanine 3-conjugated goat anti-mouse IgG (GB21301, Servicebio) and a horseradish peroxidase (HRP)-conjugated goat anti-rabbit IgG (GB23303, Servicebio). Cover slips were applied for mounting. The sections were imaged under a fluorescence microscope (Eclipse C1, Nikon, Tokyo, Japan), using a × 40 objective.

### Cytokine measurements

To determine the concentrations of granulocyte-macrophage colony-stimulating factor (GM-CSF), interferon (IFN)-γ, IL-10, IL-13, IL-17A, IL-17C, IL-17F, IL-1b, IL-4, IL-6, IL-23, and tumor necrosis factor (TNF)-α in plasma samples, we performed Meso Scale Discovery ultrasensitive multifactor electro-chemiluminescence assays (Meso Scale Discovery, Rockville, MD, USA), according to the manufacturer’s instructions. Then, plasma IL-6 concentrations and BALF IL-6 and IL-1β concentrations were measured using corresponding enzyme-linked immunosorbent assay (ELISA) kits from eBioscience (San Diego, CA, USA).

### Real-time reverse transcriptase-quantitative polymerase chain reaction (RT-qPCR) analysis

Total RNA was extracted from the lungs of mice using the TRIzol Reagent (Thermo Fisher Scientific) and reverse-transcribed into complementary DNA using a reverse transcriptase kit (Toyobo, Osaka, Japan). mRNA-expression levels were quantified using SYBR Premix EX Taq™ (TaKaRa Bio, Osaka, Japan) with β-actin expression serving as an internal control. Primers with the following sequences were used for the qPCR step: Trp63: forward: 5′-TTG TGA AAC GAT GCC CTA AC-3′; reverse: 5′-CTC TGC CTT CCC GTG ATA-3′; JAG2: forward: 5′-CCT GTG TGG TTA TCT GCG TAT-3′; reverse: 5′-GCT CTC ATC CCG TGG TAG-3′; β-actin: forward: 5′-CCT CTA TGC CAA CAC AGT-3′; reverse: 5′-AGC CAC CAA TCC ACA CAG-3′.

### Western blot analysis

Lung tissues were lysed and homogenized in radioimmunoprecipitation assay buffer with phosphatase inhibitors and protease inhibitors (Beyotime Biotechnology). Each homogenate was centrifuged at 12,000 rpm for 15 min at 4 °C to extract proteins, and the concentrations were determined by BCA protein assays (7780, CST). Equal amounts of protein from each sample were resolved by sodium dodecyl sulfate (SDS)-polyacrylamide gel electrophoresis and then transferred to polyvinylidene fluoride membranes. After blocking at room temperature for 1 h, the membranes were incubated overnight at 4 °C with antibodies against p63 (ab53039, Abcam), phosphorylated signal transducer and activator of transcription (p-STAT3; 9145, CST), STAT3 (9139, CST), jagged 2 (JAG2; NBP1-58284, Novus Biologicals, Centennial, CO, USA), and β-actin (4970, CST). After washed three times with Tris-buffered saline with Tween-20, the membranes were incubated with an appropriate HRP-conjugated secondary antibody at room temperature for 1 h. Protein bands were exposed with enhanced electro-chemiluminescence reagents (Beyotime Biotechnology), and the bands were analyzed with an Imaging System (Bio-Rad, CA, USA).

### Immunohistochemistry (IHC)

The lung sections were incubated at 56 °C for 4 h, after which they were successively immersed in xylene and 95% ethanol. To inhibit endogenous peroxidase activity, the lung sections were incubated with fresh 0.3% hydrogen peroxide in 100% methanol for 30 min at 37 °C. After washing the sections three times by PBS, antigen retrieval was performed by incubation with a 0.01 M citrate buffer (pH 6.0) at 95–100 °C for 15 min. The sections were then cooled to room temperature and washed with PBS, followed by incubation with bovine serum albumin solution for 30 min and then with an anti-p63 antibody (ab53039, Abcam; 1:300 dilution) at 37 °C for 1 h and overnight at 4 °C. Subsequently, the sections were washed three times with PBS and incubated with an HRP-conjugated goat anti-mouse secondary antibody (ab205718, Abcam) at room temperature for 45 min. The sections were imaged using a × 40 objective under a light microscope (CX43, Olympus, Tokyo, Japan).

### Cell culture and treatments

Human bronchial epithelial (HBE) cells were purchased from the American Type Culture Collection (CRL-2741), cultured in RPMI 1640 (Gibco, USA) supplemented with 10% fetal bovine serum (10082147, Gibco) and penicillin-streptomycin (100 U/ml), and maintained in 5% CO_2_ at 37 °C. After seeding in 6-wells plate for 24 h, 50 μM BLM, and 10 μg/ml MSC SLP were added, separately and simultaneously. After another 24 h, cell supernatant was collected, and total RNA and protein were extracted by TRIzol Reagent and radioimmunoprecipitation assay buffer. Short hairpin RNA (shRNA) targeting TP63 (5′-GCCACATCAAACCTTTGAGTA-3′) was introduced into the lentiviral vector, and a non-targeting sequence (5′-TTCTCCGAACGTGTCACGT-3′) was used as the negative control. After seeding in 6-wells plate for 24 h, HBE cells were infected with the lentiviral shTP63 and negative vectors.

### Protein digestion for mass spectrometry (MS) analysis

Samples were lyophilized and then lysed in SDS solution (4% SDS, 100 mM Tris, pH 7.6). The protein solution from each sample was subjected to proteolytic digestion on 10-kDa filter (Pall Life Sciences, USA) using a filter-aided sample-preparation protocol, as described in detail elsewhere [37]. The peptides in solution were transferred to a solid-phase extraction cartridge (MonoSpin C18, GL Sciences) for desalting and washing the samples. The peptides were dried by vacuum centrifugation for subsequent proteome analysis.

### Proteomic analysis

All samples obtained as described above were analyzed with a Fusion mass spectrometer (Thermo Fisher Scientific) equipped with a Nanospray Flex source (Thermo Fisher Scientific). Data-dependent acquisition was performed using Xcalibur software in profile spectrum data type. All raw Xcalibur files acquired from the MS runs were analyzed using the default settings of Proteome Discoverer 2.2 (Thermo Scientific), with minor modifications. A detailed description of data analysis is provided in the Supplementary Information section.

### Statistical analysis

Statistical analysis was performed using GraphPad Prism 8.0 software (GraphPad, San Diego, CA, USA). One-way analysis of variance (ANOVA) was performed for multiple comparisons, whereas Student’s *t* test was applied for comparisons of two groups. *P* < 0.05 was defined as the threshold for statistical significance. The results are shown as the mean ± standard deviation (SD).

## Results

### MSC SLP attenuated mortality and weight loss induced by BLM

**Fig. 1:**
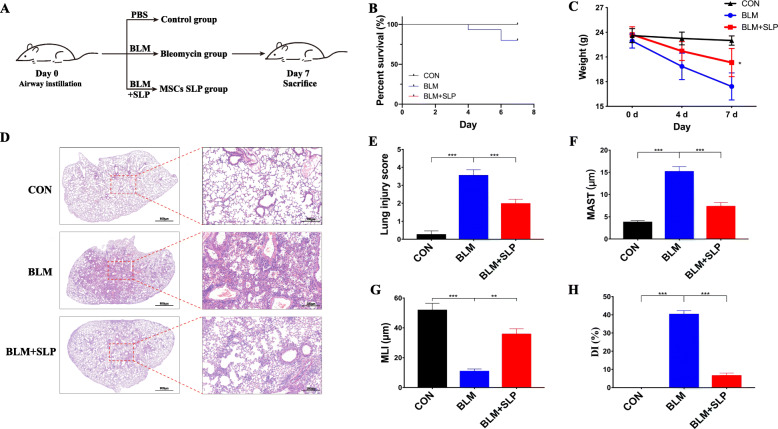
MSC SLP attenuated BLM-induced ALI in vivo. **a** Scheme representation of the mouse model established in this study. PBS, BLM (2.5 mg/kg), or BLM (2.5 mg/kg) plus MSC SLP (50 mg/kg) was intratracheally administered to mice. **b** Survival curves. Mortality of both CON and BLM + SLP group was zero. **c** Mouse weights on days 0, 4, and 7. **d** H&E staining. **e–h** Quantitative analysis of lung damage as assessed histopathologically. **e** Lung injury score. **f** Mean alveolar septal thickness (MAST). **g** Mean linear intercept (MLI). **h** Destructive index (DI). Ten fields were randomly selected for scoring. *N* = 6–8 in each group. The data shown are presented as the mean ± SD, and statistical differences were assessed by one-way ANOVA. *P < 0.05; **P < 0.01; ***P < 0.001

Intratracheal BLM has been widely used to induce ALI within 1 week in mice [36]. In this study, each mouse received 2.5 mg/kg BLM intratracheally. As shown in Fig. 1a, the mice were intratracheally administered PBS, BLM, or MSC SLP plus BLM on day 0, corresponding to the control, BLM, and MSC SLP groups, respectively. Peripheral blood, BALFs, and lung tissues were collected on day 7.

The survival status of the mice in each group was recorded daily, showing that BLM led to a higher mortality (approximately 20%) on day 7, whereas co-treatment with MSC SLP significantly reduced the mortality (0%) on day 7 (Fig. 1b). Furthermore, the mouse body weights decreased dramatically after BLM instillation, which was significantly reversed by MSC SLP (Fig. 1c). No visible weight loss was found in the control group. These results demonstrated the contribution of MSC SLP to survival and weight maintenance in the mice, suggesting its potential to exert a protective effect against ALI.

### MSC SLP attenuated BLM-induced alveolar injury

H&E staining demonstrated extensive morphological damage in BLM-instilled lungs, such as hemorrhaging, congestion, thickening of the alveolar walls, transparent membrane formation, and infiltration of inflammatory cells, especially neutrophils (Fig. 1d). In contrast, assessment of lung pathology revealed markedly more intact alveolar walls and decreased inflammation after MSC SLP treatment (Fig. 1d). Additionally, the lung-injury score (a relatively quantitative indicator) of MSC SLP-instilled mice was distinctly lower than that of BLM-instilled mice (Fig. 1e). BLM-induced alveolar septum thickening, alveolar-space broadening, and alveolar wall destruction were greatly counteracted by MSC SLP (Fig. 1f–h). No histological defects were observed in the PBS-instilled lungs (Fig. 1d–h). The above results indicated that MSC SLP played a significant role in protecting alveoli from BLM-induced damage.

### MSC SLP inhibited apoptosis induced by BLM

**Fig. 2:**
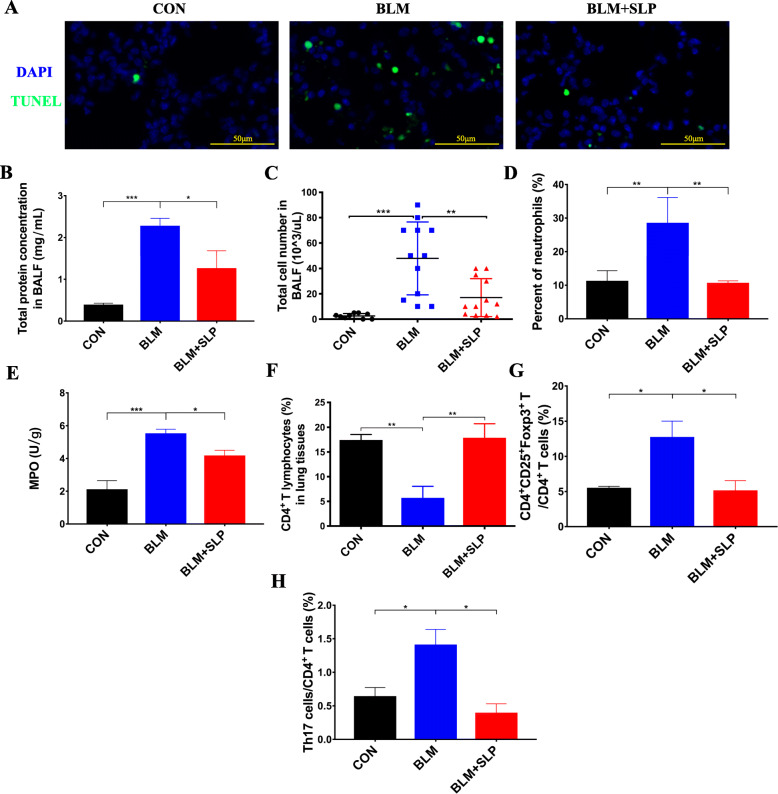
MSC SLP inhibited apoptosis and inflammatory cell infiltration induced by BLM. **a** TUNEL staining to detect apoptotic cells. **b** Total protein levels, **c** total cell counts, and **d** neutrophil percentages in BALFs were assessed. **e** MPO activities in lung homogenates were measured. **f** The percentages of CD4^+^ T cells, and **g** CD4^+^CD25^+^Foxp3^+^ Treg cells in the lungs were analyzed by flow cytometry. **h** The percentage of Th17 cells in the blood were analyzed by flow cytometry. *N* = 6–8 in each group. The data shown are presented as the mean ± SD, statistical differences were and assessed by one-way ANOVA. *P < 0.05; **P < 0.01; ***P < 0.001

TUNEL assays were performed to estimate the number of apoptotic cells containing DNA fragments during the late stages of apoptosis [38]. To clarify the effect of MSC SLP on apoptosis, we determined the degree of apoptosis in the lungs by TUNEL staining. TUNEL-positive epithelial cells dramatically increased in BLM-instilled lungs, but were remarkably reduced by MSC SLP (Fig. 2a). MSC SLP was competent in inhibiting apoptosis, which was possibly attributable to the anti-apoptotic cytokines released by MSCs.

### MSC SLP alleviated inflammatory infiltration induced by BLM

To determine the effect of MSC SLP on inflammatory infiltration, we assessed total protein levels, total cell numbers, and the profiles of inflammatory cells in BALFs. BLM instillation led to protein accumulation in BALFs, but this was remarkably blocked by MSC SLP (Fig. 2b). Moreover, BLM instillation significantly induced inflammatory cell infiltration, especially neutrophils, whereas MSC SLP significantly prevented lung inflammation (Fig. 2c, d). No statistically significant difference in inflammatory cell infiltration was found between the control and MSC SLP groups (Fig. 2c, d). To confirm this observation, the activity of MPO (a marker of neutrophilic aggregation) was evaluated in lung tissues to assess the level of neutrophils. BLM instillation caused a remarkable increase in MPO levels, which was inhibited by MSC SLP (Fig. 2e).

### MSC SLP modulated the imbalance of Treg and Th17 cells

Data from numerous studies have demonstrated that Treg and Th17 cells are associated with ALI and ARDS in both humans and mice [18–20]. Moreover, our previous study revealed that MSCs contributed to tissue repair in ALI by regulating the balance of Treg and Th17 cells [39]. We also previously illustrated the critical role of Treg and Th17 cells in recovery from ALI in mice [18]. Therefore, we examined whether MSC SLP could regulate Treg and Th17 cells in acute lung injury like MSC. Accordingly, we analyzed the balance of CD4^+^CD25^+^Foxp3^+^ Treg and Th17 cells on day 7 by flow cytometry. BLM administration led to a reduction of CD4^+^ T cells and an increased percentage of CD4^+^CD25^+^Foxp3^+^ Treg cells (Fig. 2f, g), whereas MSC SLP stimulated CD4^+^ T cell differentiation and inhibited the expansion of Treg cells in lung tissues (Fig. 2f, g). Moreover, the number of Th17 cells in the blood was significantly attenuated after MSC SLP treatment (Fig. 2h).

### MSC SLP depressed IL-6 secretion induced by BLM

**Fig. 3:**
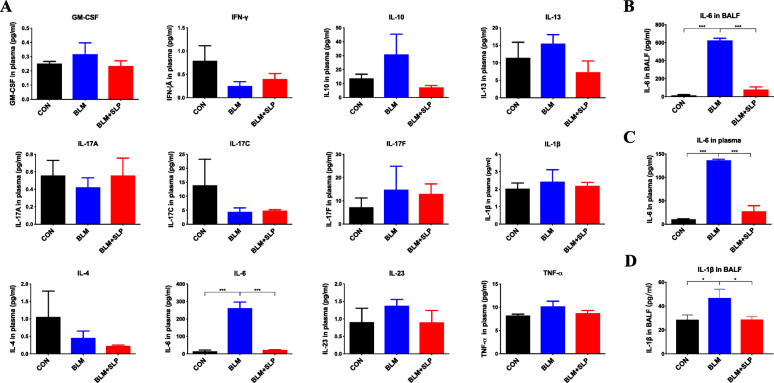
MSC SLP alleviated pro-inflammatory cytokine production induced by BLM. **a** MSD electro-chemiluminescence assays. **b**, **c** IL-6 concentrations in BALF and plasma samples were measured by ELISA. **d** IL-1β concentrations in BALFs were detected by ELISA. *N* = 6–8 in each group. The data shown are presented as the mean ± SD, and statistical differences were assessed by one-way ANOVA. *P < 0.05; ***P < 0.001

ALI is an acute and inflammatory disorder involving the release of numerous cytokines. Thus, multiplex cytokine-detection technology was applied. Among all cytokines detected in the plasma, the IL-6 concentration was elevated by BLM and efficiently mitigated by MSC SLP (Fig. 3a). To verify these findings, the levels of IL-6 in plasma and BALFs were also measured by ELISA. MSC SLP markedly neutralized IL-6 induction by BLM in both plasma and BALFs (Fig. 3b, c). ELISA also showed that MSC SLP attenuated the increased IL-1β production in BALFs (Fig. 3d). MSC SLP tended to lower the levels of IL-10, IL-13, and IL-23 in the plasma, although these differences were not statistically significant (Fig. 3a).

### Lyophilized powder of MSC-free medium exhibited non-protective function against BLM-induced ALI

Histopathology analysis, including H&E staining and lung injury score, demonstrated that lyophilized powder of MSC-free medium (BLM + M group) had no protective effect on BLM-induced acute lung injury (Supplementary Fig. 2a–e). Total protein levels, total cell numbers, and the percent of neutrophils in BALF suggested no statistical difference between mice instilled with BLM with and without MSC-free medium (Supplementary Fig. 2f–h). Moreover, MSC-free medium showed no inhibitory effect on the secretion of IL-6 and IL-1β induced by BLM (Supplementary Fig. 2i, j). These inflammatory indicators suggested that lyophilized powder of MSC-free medium did not prevent inflammatory infiltration, and the repair capacity of MSC SLP in BLM-induced ALI was attributed to the secretome from MSC instead of MSC-free medium.

### MSC SLP activated p63 by inhibiting IL-6–p-STAT3 signaling

**Fig. 4:**
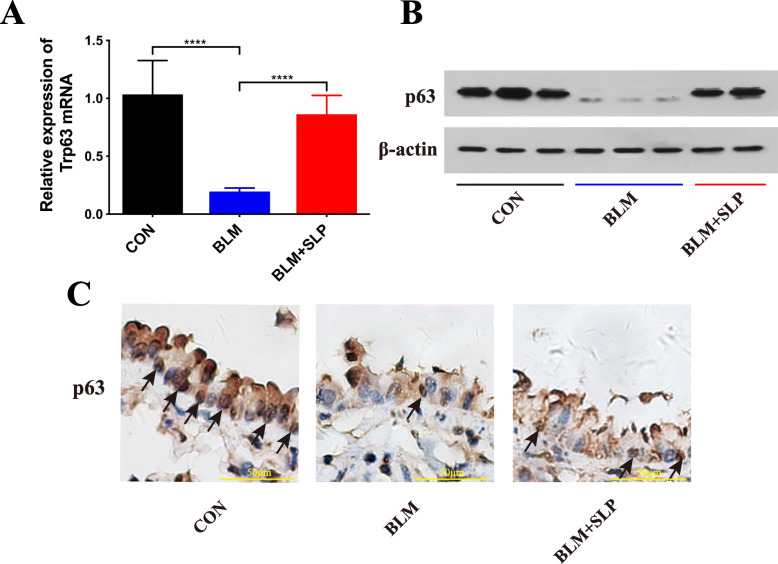
MSC SLP increased p63 expression. The expression of p63 in lung tissues was detected by **a** RT-qPCR, **b** western blotting, and **c** IHC. Arrows indicate p63^+^ cells. *N* = 6–8 in each group. The data shown are presented as the mean ± SD, and statistical differences were assessed by one-way ANOVA. ****P < 0.0001

**Fig. 5:**
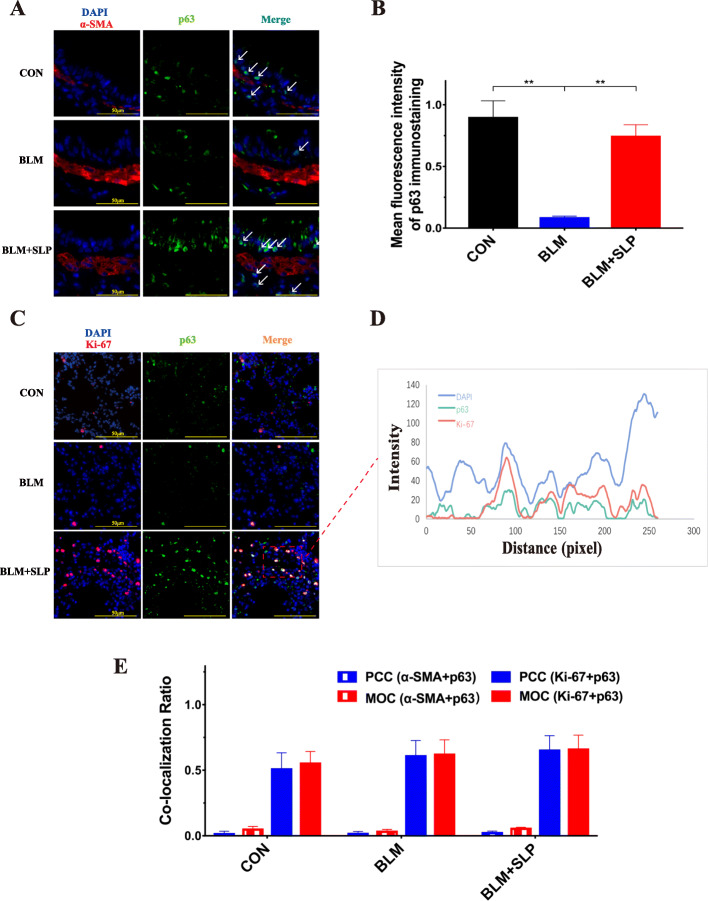
MSC SLP promoted p63^+^ cell proliferation in the lungs. **a** Immunofluorescence staining with antibodies against p63 and α-SMA. **b** Quantitative analysis of p63 expression, as determined by immunofluorescence staining. **c** Immunofluorescence staining with antibodies against p63 and Ki-67. **d** Staining-intensity profiles showing signals from all three fluorescent channels. **e** PCC and MOC values. Ten fields were randomly selected for scoring. The data shown are presented as the mean ± SD, and statistical differences were assessed by one-way ANOVA. **P < 0.01

p63 has been shown to support self-renewal, inhibit cell apoptosis, and help maintain homeostasis in epithelial cells [28, 30–32]. In this study, RT-qPCR and western blot analysis revealed that p63 expression dramatically decreased in BLM-instilled mice and that MSC SLP restored p63 expression (Fig. 4a, b). In agreement, IHC showed that p63 was highly expressed in the basal layers of the airways, but was not expressed in the muscle layer of the airways. p63 expression decreased in BLM-instilled mice, but was partially recovered after MSC SLP treatment (Fig. 4c). Furthermore, immunofluorescence staining also showed that p63 expression was re-activated by MSC SLP (Fig. 5a–c). α-SMA, a marker of airway smooth muscle cells in the lungs, did not co-localize with p63 (Fig. 5a, e).

Ki-67 (a proliferation marker) markedly co-localized with p63, especially in MSC SLP-instilled lungs (Fig. 5c–e), indicating that a considerable portion of p63^+^ cells was actively proliferating and repairing damage after MSC SLP instillation. Intensity profiles showed various degrees of co-localization between p63 and Ki-67 (Fig. 5d). To quantify the degree of co-localization between the fluorophores, Pearson’s correlation coefficient (PCC) and Mander’s overlap coefficient (MOC) [40] values were determined. Quantitative analysis verified that the p63 protein did not co-localize with α-SMA in the lungs, but was highly co-localized with Ki-67 (Fig. 5e).

**Fig. 6:**
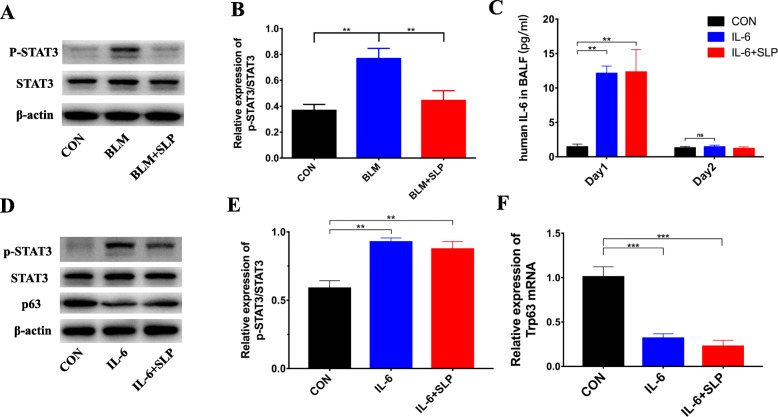
MSC SLP inhibited STAT3 phosphorylation, and rh IL-6 enhanced STAT3 phosphorylation to reduce p63 expression. **a** The levels of p-STAT3, STAT3, and β-actin proteins were measured by western blotting. **b** Quantitative analysis of p-STAT3 expression. **c** The concentrations of rh IL-6 in the lungs on days 1 and 2 after rh IL-6 administration. **d** The protein levels of p-STAT3, STAT3, p63, and β-actin were measured by western blotting. **e** Quantitative analysis of p-STAT3 expression. **f** p63 mRNA-expression levels were measured by RT-qPCR. *N* = 6–8 in each group. The data shown are presented as the mean ± SD, and statistical differences were assessed by one-way ANOVA. **P < 0.01; ***P < 0.001

Previous data showed that IL-6 regulates STAT3 signaling [41]. Therefore, we subsequently detected STAT3 and phosphorylated STAT3 (p-STAT3) by western blotting. STAT3 was evidently activated to p-STAT3 in BLM-induced ALI (Fig. 6a, b). In contrast, MSC SLP significantly inhibited STAT3 phosphorylation (Fig. 6a, b). Considering that IL-6 appeared to promote STAT3 phosphorylation in basal cells of the airways and previous data showed that the IL-6–p-STAT3 pathway regulated p63 isoform expression in keratinocytes [41, 42], we hypothesized that a sharp rise of IL-6 boosted STAT3 phosphorylation and then restrained p63 expression in BLM-induced ALI. Our results suggested that MSC SLP activated p63 by inhibiting the IL-6–p-STAT3 pathway.

### Intratracheal rh IL-6 reduced p63 expression

To clarify the role of IL-6, we instilled rh IL-6 into the airways of mice. ELISA demonstrated that rh IL-6 was enriched in the lung tissues on day 1, after which it was absorbed and removed on day 2 (Fig. 6c). Consistently, rh IL-6 administration promoted STAT3 phosphorylation and decreased p63 expression (Fig. 6d–f). In the presence of sufficient exogenous IL-6, MSC SLP no longer re-activated p63 expression after rh IL-6 instillation. These results suggested that MSC SLP alleviated ALI by inhibiting inflammatory cell recruitment and reducing IL-6 production without blocking IL-6 function.

### MSC SLP increased JAG2 expression

**Fig. 7:**
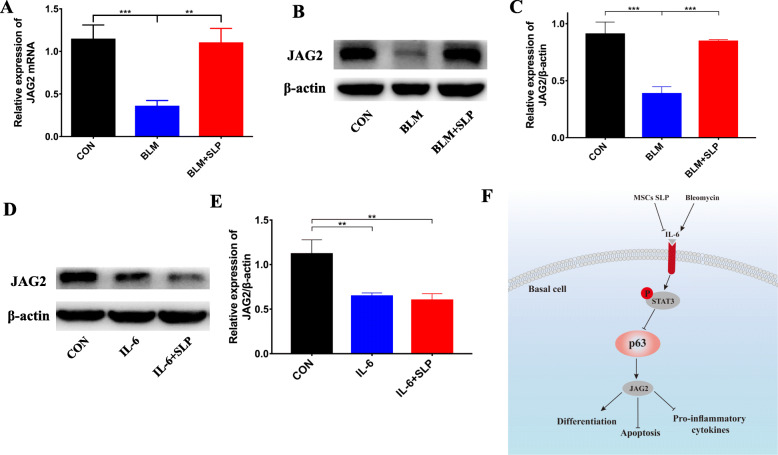
MSC SLP increased JAG2 expression by activating p63. **a** JAG2 mRNA-expression levels in a mouse model of ALI. **b** The protein-expression levels of JAG2 and β-actin in a mouse model of ALI. **c** Quantitative analysis of JAG2 expression. **d** The protein-expression levels of JAG2 and β-actin in mice treated with IL-6 ± SLP. **e** Quantitative analysis of JAG2 expression. **f** Schematic model of how MSC SLP attenuates BLM-induced ALI. *N* = 6–8 in each group. The data shown are presented as the mean ± SD, and statistical differences were assessed by one-way ANOVA. **P < 0.01; ***P < 0.001

To clarify the mechanism whereby MSC SLP alleviated ALI, we tested various target genes of p63, especially those linked to basal cell function, such as JAG2. To validate the role of p63 in regulating JAG2 expression, we also demonstrated that TP63 silencing apparently downregulated the expression of JAG2 at both the mRNA and protein levels, in vitro (Supplementary Fig. 3a–c). Here, JAG2 was illustrated to be a down-stream target of p63. In BLM-instilled lungs, the mRNA and protein levels of JAG2 were both significantly downregulated by p63 inhibition (Fig. 7a–c), whereas MSC SLP greatly increased JAG2 expression (Fig. 7a–c). Administration of rh IL-6 reduced JAG2 expression, which was not reversed by MSC SLP (Fig. 7d, e). A schematic model of the mechanism whereby MSC SLP alleviated BLM-induced ALI is proposed in Fig. 7f.

### MSC SLP activated p63 to promote cell survival in vitro

To illustrate the role of MSC SLP in cell survival and apoptosis in vitro, BLM and MSC SLP were applied to HBE cells, separately and simultaneously. TUNEL staining was preformed to evaluate the degree of apoptosis in HBE cells. TUNEL-positive cells were dramatically increased by BLM and remarkably reduced by MSC SLP (Supplementary Fig. 4a, b), demonstrating the role of MSC SLP in inhibiting cell apoptosis. Moreover, BLM was revealed to decrease cell viability and induce the cleavage of caspase-3, while MSC SLP was found to promote cell survival and inhibit cleavage of caspase-3 (Supplementary Fig. 4c-e). ELISA showed that MSC SLP significantly prohibited IL-6 production and secretion induced by BLM (Supplementary Fig. 4f). Consistently, RT-qPCR revealed that BLM significantly increased IL-6 in mRNA level, and MSC SLP significantly inhibited the increase of IL-6 transcription (Supplementary Fig. 4g). Furthermore, MSC SLP attenuated the phosphorylation of STAT3 induced by BLM and re-activated the expression of p63 and JAG2 at both the mRNA and protein levels (Supplementary Fig. 4h-j).

## Discussion

Preclinical studies and currently ongoing clinical trials have shown that MSC administration has the potential to become an effective therapeutic strategy for treating ALI [12, 13, 16, 43–45]. MSCs exert protective effects against ALI either by differentiating into alveolar epithelial cells after damage or by secreting soluble factors such as keratinocyte growth factors, anti-inflammatory cytokines, and extracellular vesicles [16, 44, 45]. However, limitations in terms of delivery and risks of instability in humans are both concerns, especially considering the danger of iatrogenic tumor formation [16]. The secretome of MSCs, composed of extracellular vesicles and released bioactive molecules, is a viable alternative cell-free therapy. MSC-conditioned media, containing proteins, cytokines, and chemokines released by MSCs, can enhance calvarial bone regeneration in rats [12] and induce neutrophil apoptosis to attenuate ALI [46]. The secretome of MSCs can be purified and freeze-dried into lyophilized powder to achieve large-scale production, easier storage, and stable bioactivity for a long time [47]. To delineate the bioactive ingredients in lyophilized powder from MSCs, liquid chromatography (LC)-tandem MS technology was performed, and numerous active biomolecules potentially involved are shown in Supplementary Table 1.

In this study, MSC SLP was found to reduce mortality, help maintain alveolar morphological structures, reduce alveolar inflammation, and inhibit epithelial cell apoptosis in BLM-induced ALI. The induction of p63 expression by MSC SLP was the most important finding of our study. Data from numerous studies have demonstrated that p63, a homolog of p53, is vital for the normal development and homeostasis of epithelial tissues in both humans and mice. For instance, heterozygous mutations in human p63 drive several developmental defects and disorders, especially skin abnormalities [48–50]. In animal studies, p63 knockout led to severe anomalies in the development of epithelia and their derivatives, and even death at birth [32, 51]. p63 is also widely recognized to be involved in tumorigenesis, especially epithelial tumors, such as those associated with prostate, bladder, and colorectal cancers [33, 34]. In addition, p63 is a specific marker of basal cells and performs a vital role in helping maintain self-renewal potential and inhibit apoptosis in various epithelial cells, including those in the mouse trachea and human airways [28, 29, 31]. In this study, we showed that MSC SLP re-activated p63 expression and suppressed epithelial cell apoptosis in BLM-induced ALI, indicating that p63 plays a crucial role in ALI development. Immunofluorescence staining against p63 and Ki-67 demonstrated the presence of proliferating p63^+^ cells. Thus, MSC SLP was verified to activate p63 expression to improve the proliferative capacity and inhibit apoptosis in progenitor cells.

IL-6 is a pleiotropic cytokine that is produced in response to various inflammatory stimuli and that is associated with multiple inflammatory disorders of the lungs [22–25]. In BLM-treated mice, IL-6 promoted inflammatory infiltration at an early inflammatory stage [27]. In this study, on day 7, the sharp increase of IL-6 production in response to BLM stimulation was markedly repressed by MSC SLP. Moreover, IL-6 appeared to regulate p63 expression by promoting STAT3 phosphorylation in mice. Previous data revealed that the IL-6–p-STAT3 pathway was activated during SO_2_-induced airway injury in mice and that STAT3 activation in basal cells regulated ciliogenesis through tyrosine phosphorylation [41]. IL-6–p-STAT3-pathway activation modulated expression of the p63 isoform in keratinocytes during regeneration associated with wound healing [42]. In this study, we found that hyperphosphorylation of STAT3, attributed to the excessive IL-6 secretion, inhibited p63 expression in BLM-induced ALI, whereas MSC SLP increased p63 expression by suppressing excessive activation of IL-6 and p-STAT3.

CD4^+^CD25^+^Foxp3^+^ Treg cells negatively regulate immune and inflammatory responses by cell-contact-dependent suppression and secretion of inhibitory cytokines, such as IL-10 and transforming growth factor (TGF)-β, which prevents chronic immunopathology after pathogen clearance [17]. However, findings from studies on the roles of Treg cells in different diseases seem contradictory [52]. Under many inflammatory conditions, a sudden increase of Treg cells without beneficial effects can occur [52]. For instance, patients with ALI or ARDS often show increased levels of Treg cells, which have been associated with the mortality of patients with ARDS [19, 20]. In this study, BLM increased the percentage of CD4^+^CD25^+^Foxp3^+^ Treg cells in the lungs, which was reversed by MSC SLP. Apparently, the surge in Treg cell production after BLM instillation could not control inflammatory injury. Th17 cells have opposite roles to Treg cells and represent a subset of pro-inflammatory cells [17]. In ARDS, Th17 cells enhanced the accumulation of pro-inflammatory cytokines and amplified inflammatory responses [19, 20]. Here, we found that MSC SLP dramatically attenuated the increase of Th17 cells in the blood. We previously demonstrated in vivo that MSCs decreased Th17 cell differentiation to promote recovery from ALI and inhibition of Th17 cells using IL-17 or IL-22 antibodies also alleviated ALI by inhibiting the recruitment of neutrophils and macrophages [18, 39]. Consistent with the in vivo study, MSCs were also found to inhibit the differentiation of naive CD4^+^ T cells into Th17 cells in vitro [53]. The results suggested that MSC SLP could partially, at least, simulate the role of MSCs in decreasing Th17 cells differentiation to protect against ALI.

Furthermore, accumulating evidence has revealed that IL-6 can synergize with TGF-β to initiate Th17 polarization by promoting STAT3 phosphorylation [54–56]. Consistently, we found that MSC SLP strongly inhibited IL-6 production and STAT3 phosphorylation. Our previous study revealed the critical role of Th17 cells in inhibiting inflammatory response and promoting recovery from ALI in mice [18]. Therefore, we hypothesize that MSC SLP restrained Th17 cell expansion by inhibiting IL-6-induced STAT3 phosphorylation, which eventually alleviated acute inflammatory injury. However, the distinct mechanism underlying MSC SLP and Th17 cells still needs further study.

p63-mediated target gene expression confers stem cell properties, such as proliferative capacity and differentiation, in basal cells [30, 31]. Previous findings revealed that the expression of JAG2 (a Notch ligand) was directly regulated by p63 in epithelia, which was associated with progenitor cell differentiation and restrained inflammation. In vitro, JAG2 was strongly expressed on the surface of p63^+^ adult airway progenitor cells [57]. In vivo, JAG2 expression was enhanced by p63 and was required for thymic development [58]. JAG2-knockout mice presented with limb defects and thymic underdevelopment, and some even died at birth like p63-knockout mice [59]. Increased JAG2 expression in p63^+^ airway progenitor cells could activate Notch3 signaling in neighboring cells, ultimately promoting p63^+^ cell expansion as well as the transition to early epithelial progenitors and later to daughter cell fate selection [57, 60, 61]. Moreover, JAG2 inhibited monocyte recruitment by reducing the expression of inflammation-related genes in human non-small cell lung cancer cells [62]. Consistently, we observed that JAG2 expression was downregulated by reduced p63 signaling in BLM-instilled lungs and was upregulated by MSC SLP treatment. Therefore, we postulate that MSC SLP activated the p63–JAG2 pathway, which alleviated inflammatory cell recruitment and promoted the repair of damaged epithelial tissue.

Emerging evidence has illustrated that lyophilization does not disturb the structure and integrity of extracellular vesicles [63] and does not weaken the stability of proteins and lipid components [47]. Airway administration of MSC SLP ensured that it was relatively enriched at sites of damage in the lungs, suggesting that MSC SLP holds substantial therapeutic promise for clinical applications.

## Conclusions

We demonstrated that MSC SLP inhibited the IL-6–p-STAT3 signaling pathway and then activated p63–JAG2 signaling to promote epithelial cell proliferation, inhibit cell apoptosis, reduce inflammatory cell recruitment, and ameliorate ALI. Inhibition of the IL-6–p-STAT3-signaling pathway also restricted Th17 cell polarization, contributing to the suppression of the inflammatory response. The results of this study shed light on the mechanisms of BLM-induced ALI and suggest that treatment with MSC SLP is a promising therapeutic approach.

## Supplementary Information

**Additional file 1: Supplementary Fig. 1.** The overall procedure used for MSC SLP production. Supernatant from placenta-derived MSCs was collected in vials (300 μl/vial). The following optimized procedure and parameters were used: pre-freezing (− 45 °C, 240 min, normal pressure), primary drying (− 40 °C, 3300 min, vacuum of 0 Pa), and secondary drying (30 °C, 180 min, vacuum of 20 Pa).

**Additional file 2: Supplementary Fig. 2.** Lyophilized powder of MSC-free medium could not repair BLM-induced ALI and inhibit inflammatory infiltration. a H&E staining. b-e Quantitative analysis of lung damage as assessed histopathologically. Ten fields were randomly selected for scoring. b Lung injury score. c Mean alveolar septal thickness (MAST). d Mean linear intercept (MLI). e Destructive index (DI). f Total protein levels g total cell counts, and h neutrophil percentages in BALFs were assessed. i IL-6 and j IL-1β concentrations in BALFs were detected by ELISA. *N* = 6–8 in each group. The data shown are presented as the mean ± SD, and statistical differences were assessed by one-way ANOVA. *P < 0.05; **P < 0.01; ***P < 0.001

**Additional file 3: Supplementary Fig. 3.** TP63 silencing apparently downregulated the expression of JAG2. The expression of **a** TP63 and **b** JAG2 in HBE cells was detected by RT-qPCR. **c** The protein levels of p63 and JAG2 in HBE cells were detected by western blotting.

**Additional file 4: Supplementary Fig. 4.** MSC SLP activated p63 to promote cell survival in vitro. a TUNEL staining to detect apoptotic cells. b Percent of TUNEL-positive cells. c-d Cell viability detected by Cell Counting Kit-8. e The levels of total caspase-3 and cleaved caspase-3 were measured by western blotting. IL-6 f protein level in cell supernatant and g mRNA level. The expression of h TP63 and i JAG2 was detected by RT-qPCR. j The protein levels of p-STAT3, STAT3, p63, JAG2 and β-actin were measured by western blotting. *N* = 3–4 in each group. The data shown are presented as the mean ± SD, and statistical differences were assessed by one-way ANOVA. *P < 0.05; **P < 0.01; ***P < 0.001.

**Additional file 5: Supplementary Table 1.** Bioactive ingredients in MSC SLP detected by LC-MS/MS. Proteins was digested and extracted form MSC SLP and analyzed by MS. Various biomolecules were identified in MSC SLP, based on the MS data and analysis with Proteome Discoverer software (version 2.2).

## Data Availability

All data needed to evaluate the conclusions in the paper are present in the paper and/or the Supplementary Materials. Additional data available from authors upon request.

## Abbreviations

α-SMA: Alpha smooth muscle actin
ALI: Acute lung injury
ANOVA: Analysis of variance
APC: Allophycocyanin
ARDS: Acute respiratory distress syndrome
BALF: Bronchoalveolar lavage fluid
BCA: Bicinchoninic acid
BLM: Bleomycin
CD: Cluster of differentiation
CST: Cell Signaling Technology
DI: Destructive index
ELISA: Enzyme-linked immunosorbent assay
FITC: Fluorescein isothiocyanate
Foxp3: Forkhead box P3
GM-CSF: Granulocyte-macrophage colony-stimulating factor
HBE: Human bronchial epithelial
H&E: Hematoxylin and eosin
HRP: Horseradish peroxidase
IHC: Immunohistochemistry
IFN: Interferon
IL: Interleukin
JAG2: Jagged 2
Krt14: Cytokeratin 14
Krt5: Cytokeratin 5
LC: Liquid chromatography
MAST: Mean alveolar septal thickness
MLI: Mean linear intercept
MS: Mass spectrometry
MOC: Mander’s overlap coefficient
MSC: Mesenchymal stem cell
MPO: Myeloperoxidase
P1: Passage 1
p63: Tumor protein 63
p-STAT: Phosphorylated signal transducer and activator of transcription
PBS: Phosphate-buffered saline
PCC: Pearson’s correlation coefficient
rh: Recombinant human
SD: Standard deviation
SDS: Sodium dodecyl sulfate
shRNA: Short hairpin RNA
SLP: Supernatant lyophilized powder
TGF: Transforming growth factor
TH17: T helper 17
TNF: Tumor necrosis factor
Treg: Regulatory T cell
TUNEL: Terminal deoxynucleotidyl transferase dUTP nick-end labeling
