# Supplementary figures and images for: Lyophilized powder of mesenchymal stem cell supernatant attenuates acute lung injury through the IL-6–p-STAT3–p63–JAG2 pathway
Source: Stem Cell Res Ther. 2021 Mar 29;12:216. doi: 10.1186/s13287-021-02276-y (PMC8008635; doi:10.1186/s13287-021-02276-y)

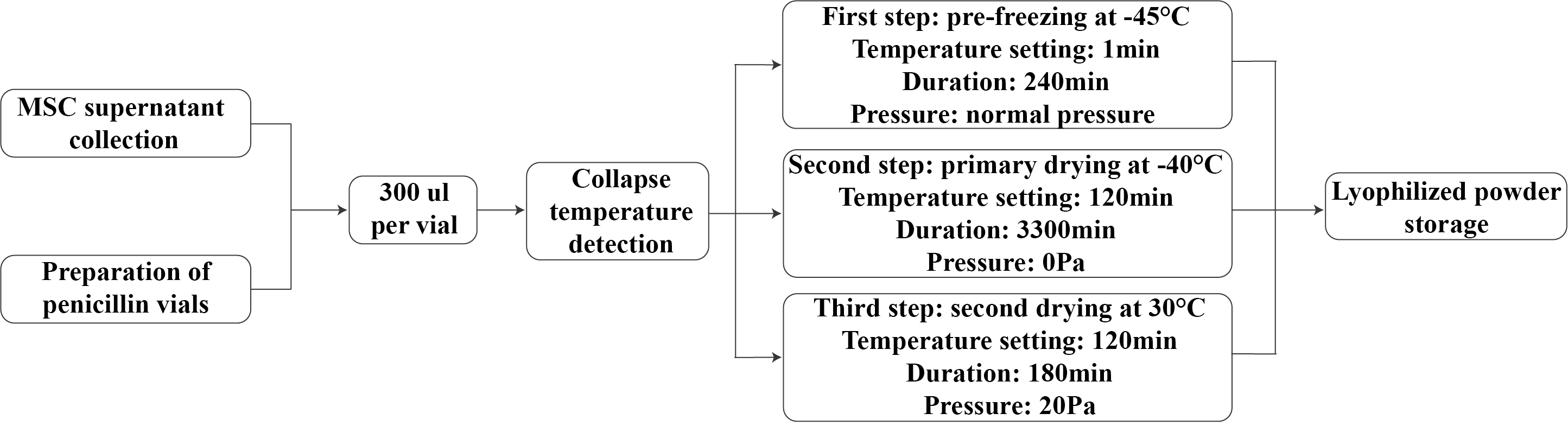

Supplement: Supplementary file 1 — Additional file 1: Supplementary Fig. 1. The overall procedure used for MSC SLP production. Supernatant from placenta-derived MSCs was collected in vials (300 μl/vial). The following optimized procedure and parameters were used: pre-freezing (− 45 °C, 240 min, normal pressure), primary drying (− 40 °C, 3300 min, vacuum of 0 Pa), and secondary drying (30 °C, 180 min, vacuum of 20 Pa). [file 13287_2021_2276_MOESM1_ESM.tif]

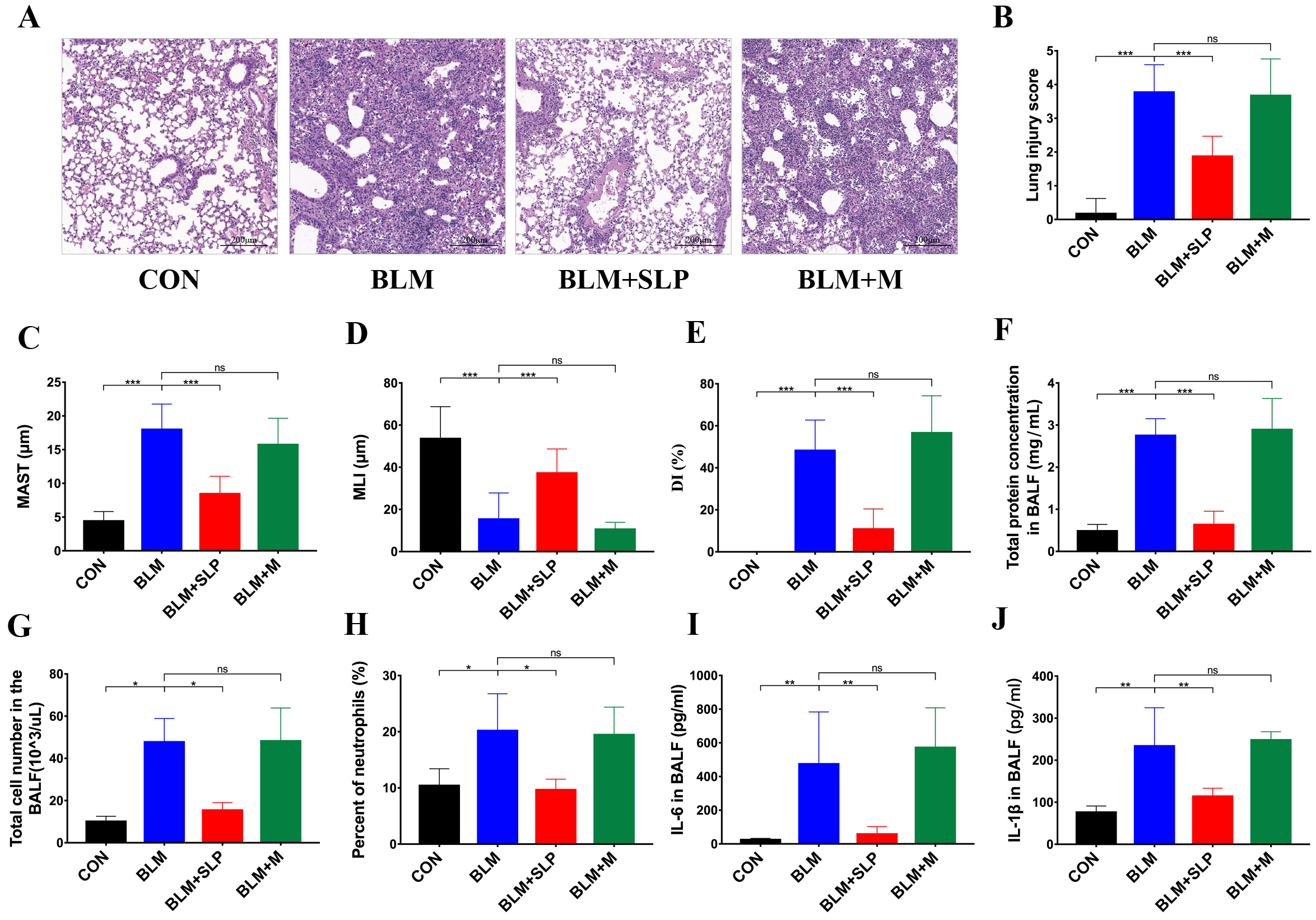

Supplement: Supplementary file 2 — Additional file 2: Supplementary Fig. 2. Lyophilized powder of MSC-free medium could not repair BLM-induced ALI and inhibit inflammatory infiltration. a H&E staining. b-e Quantitative analysis of lung damage as assessed histopathologically. Ten fields were randomly selected for scoring. b Lung injury score. c Mean alveolar septal thickness (MAST). d Mean linear intercept (MLI). e Destructive index (DI). f Total protein levels g total cell counts, and h neutrophil percentages in BALFs were assessed. i IL-6 and j IL-1β concentrations in BALFs were detected by ELISA. N = 6–8 in each group. The data shown are presented as the mean ± SD, and statistical differences were assessed by one-way ANOVA. *P < 0.05; **P < 0.01; ***P < 0.001 [file 13287_2021_2276_MOESM2_ESM.tif]

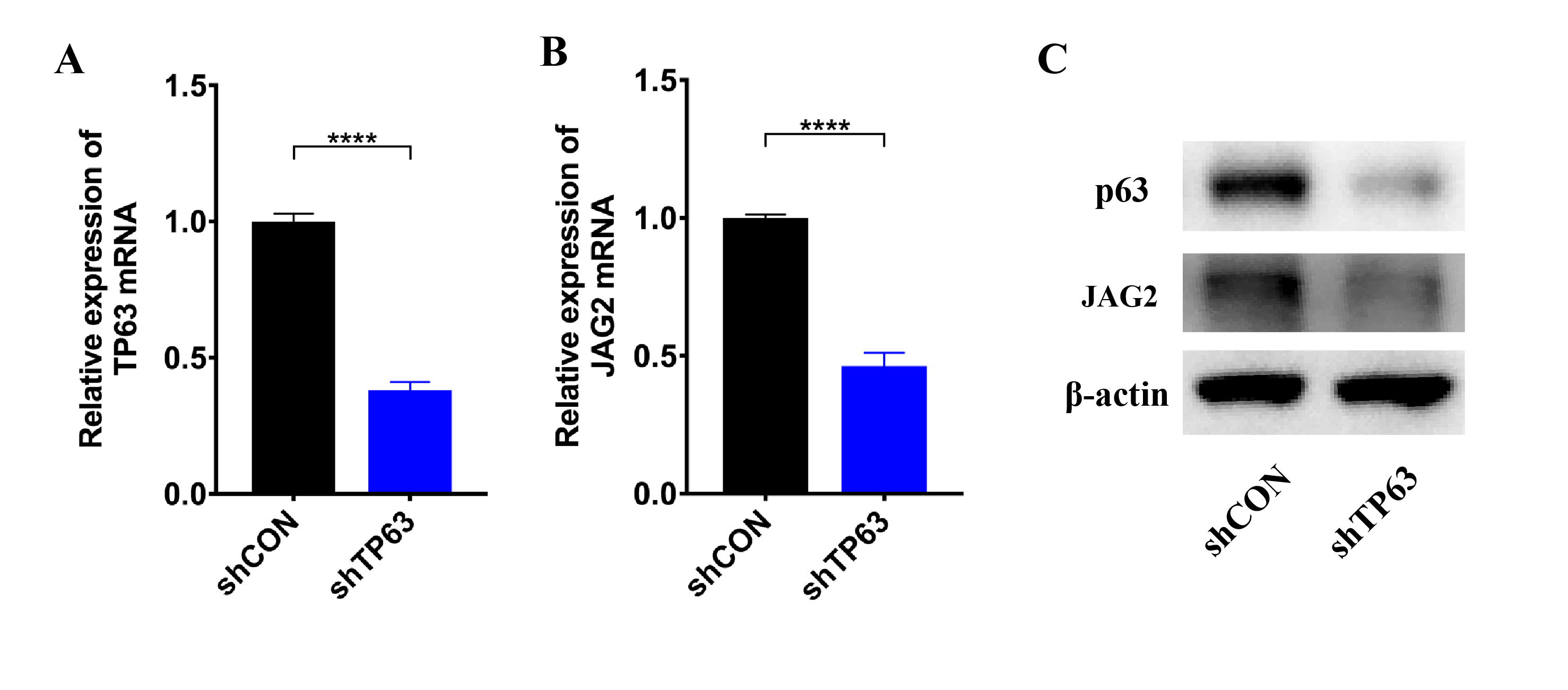

Supplement: Supplementary file 3 — Additional file 3: Supplementary Fig. 3. TP63 silencing apparently downregulated the expression of JAG2. The expression of a TP63 and b JAG2 in HBE cells was detected by RT-qPCR. c The protein levels of p63 and JAG2 in HBE cells were detected by western blotting. [file 13287_2021_2276_MOESM3_ESM.tif]

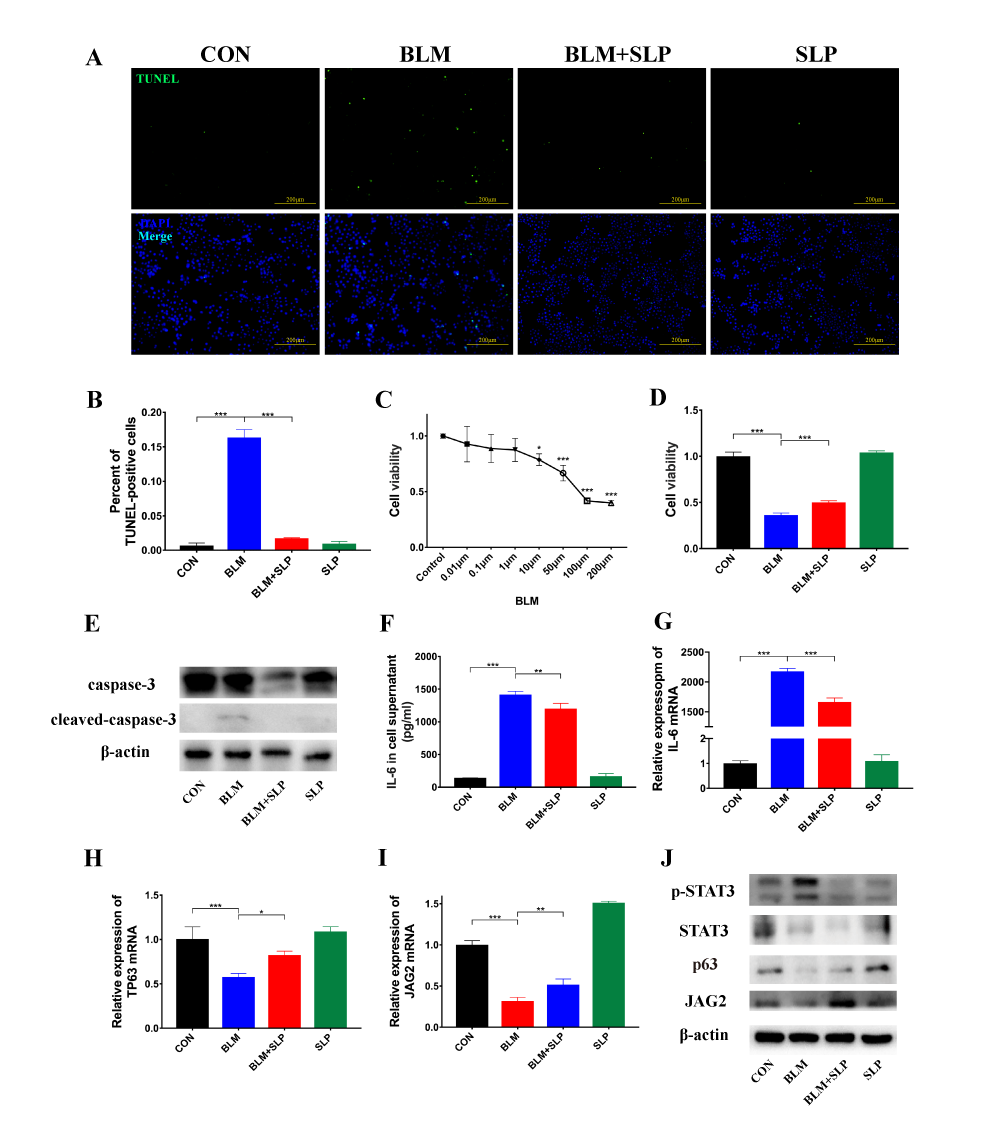

Supplement: Supplementary file 4 — Additional file 4: Supplementary Fig. 4. MSC SLP activated p63 to promote cell survival in vitro. a TUNEL staining to detect apoptotic cells. b Percent of TUNEL-positive cells. c-d Cell viability detected by Cell Counting Kit-8. e The levels of total caspase-3 and cleaved caspase-3 were measured by western blotting. IL-6 f protein level in cell supernatant and g mRNA level. The expression of h TP63 and i JAG2 was detected by RT-qPCR. j The protein levels of p-STAT3, STAT3, p63, JAG2 and β-actin were measured by western blotting. N = 3–4 in each group. The data shown are presented as the mean ± SD, and statistical differences were assessed by one-way ANOVA. *P < 0.05; **P < 0.01; ***P < 0.001. [file 13287_2021_2276_MOESM4_ESM.tif]
