# Supplementary material for: Lyophilized powder of mesenchymal stem cell supernatant attenuates acute lung injury through the IL-6–p-STAT3–p63–JAG2 pathway
Source: Stem Cell Res Ther. 2021 Mar 29;12:216. doi: 10.1186/s13287-021-02276-y (PMC8008635; doi:10.1186/s13287-021-02276-y)
Supplement: Supplementary file 5 — Additional file 5: Supplementary Table 1. Bioactive ingredients in MSC SLP detected by LC-MS/MS. Proteins was digested and extracted form MSC SLP and analyzed by MS. Various biomolecules were identified in MSC SLP, based on the MS data and analysis with Proteome Discoverer software (version 2.2). [file 13287_2021_2276_MOESM5_ESM.docx]

| **Supplementary Table 1. Proteins identified in MSC SLP** | | | |
| --- | --- | --- | --- |
| **Protein names** | **Gene names** | **Protein names** | **Gene names** |
| Epidermal growth factor receptor | EGFR | CD44 antigen | CD44 |
| Tissue factor pathway inhibitor | TFPI | Plasminogen activator inhibitor 1 | SERPINE1 |
| Dickkopf-related protein 1 | DKK1 | CD5 antigen-like | CD5L |
| Heparin cofactor 2 | SERPIND1 | Filamin-A | FLNA |
| Insulin-like growth factor-binding protein 2 | IGFBP2 | Insulin-like growth factor-binding protein 3 | IGFBP3 |
| Insulin-like growth factor-binding protein 5 | IGFBP5 | Insulin-like growth factor-binding protein 7 | IGFBP7 |
| Transgelin-2 | TAGLN2 | Tissue factor pathway inhibitor 2 | TFPI2 |
